# Acute aquatic toxicity of tire and road wear particles to alga, daphnid, and fish

**DOI:** 10.1007/s10646-011-0750-x

**Published:** 2011-07-26

**Authors:** Christopher Marwood, Britt McAtee, Marisa Kreider, R. Scott Ogle, Brent Finley, Len Sweet, Julie Panko

**Affiliations:** 1 ChemRisk Canada, 291 Woodlawn Road West, Guelph, ON N1H 7L6 Canada; 2 ChemRisk, 20 Stanwix Street, Suite 505, Pittsburgh, PA 15222 USA; 3 Pacific EcoRisk, Inc., 2250 Cordelia Road, Fairfield, CA 94534 USA; 4 ChemRisk, 25 Jessie Street, Suite 1800, San Francisco, CA 94105 USA; 5 Present Address: NovaTox Inc., 10 Crane Avenue, Guelph, ON N1G 2R2 Canada; 6 Present Address: School of Public Health, University of Michigan, 1415 Washington Heights, Ann Arbor, MI 48109 USA

**Keywords:** Tire, Particle, *Daphnia*, *Pimephales*, Sediment, Toxicity

## Abstract

Previous studies have indicated that tire tread particles are toxic to aquatic species, but few studies have evaluated the toxicity of such particles using sediment, the likely reservoir of tire wear particles in the environment. In this study, the acute toxicity of tire and road wear particles (TRWP) was assessed in *Pseudokirchneriella subcapita*, *Daphnia magna*, and *Pimephales promelas* using a sediment elutriate (100, 500, 1000 or 10000 mg/l TRWP). Under standard test temperature conditions, no concentration response was observed and EC/LC_50_ values were greater than 10,000 mg/l. Additional tests using *D. magna* were performed both with and without sediment in elutriates collected under heated conditions designed to promote the release of chemicals from the rubber matrix to understand what environmental factors may influence the toxicity of TRWP. Toxicity was only observed for elutriates generated from TRWP leached under high-temperature conditions and the lowest EC/LC_50_ value was 5,000 mg/l. In an effort to identify potential toxic chemical constituent(s) in the heated leachates, toxicity identification evaluation (TIE) studies and chemical analysis of the leachate were conducted. The TIE coupled with chemical analysis (liquid chromatography/mass spectrometry/mass spectrometry [LC/MS/MS] and inductively coupled plasma/mass spectrometry [ICP/MS]) of the leachate identified zinc and aniline as candidate toxicants. However, based on the high EC/LC_50_ values and the limited conditions under which toxicity was observed, TRWP should be considered a low risk to aquatic ecosystems under acute exposure scenarios.

## Introduction

Road dust has become a topic of interest, particularly in relation to ecological impacts on aquatic environments, which are believed to be the ultimate repository for road dust in the environment as a result of roadway runoff. Tire and road wear particles (TRWP) are among many constituents (including brake and clutch wear and corrosion particles from vehicle components and road structures) of road dust and are formed by the rolling shear of tread against the road surface. As a result of the interaction of tire particles with road surface, TRWP consist of a complex mixture of rubber, with both embedded asphalt and minerals from the pavement, as well as free pavement. Although true TRWP are difficult to collect in isolation, Kreider et al. (2010) recently provided a thorough characterization of the morphological and chemical properties of TRWP, which revealed a distinct shape, size and composition resulting from the wear mechanism created at the tire-pavement interface. The TRWP were shown to be markedly different from a tread particle (TP) generated through laboratory mechanical abrasion of the tread or grinding of tread pieces.

The toxicity of TP in aquatic species has been evaluated and attributed to both inorganic and organic constituents of tires, however, most toxicological studies to date have focused on TP generated from shredded/powdered tire tread or extracts of tire tread (Gualtieri et al. 2005a; Henkelmann et al. 2001; Wik 2007; Wik and Dave 2005, 2006; Zheng et al. 2002). Zinc has been identified as an agent causing toxicity in several studies (Gualtieri et al. 2005b; Nelson et al. 1994; Wik et al. 2009), although the release of zinc from TP has been shown to vary based on surface area (particle size), loading rates, and extraction conditions (Gualtieri et al. 2005b). While zinc can be leached from TP in the laboratory, under real world conditions, the presence of TP in a soil or sediment matrix increases the pH, which significantly hinders the leaching of Zn and movement into the pore water (Smolders and Degryse 2002; European Commission 2008).

In another study, estimates of median toxic effect levels for tire particles in aquatic organisms were reported to range from 100 to 100,000 mg/l (Wik et al. 2009). The variable toxicity of TP may be due in part to the tire formula (Wik and Dave 2005), but is also related to the methods used to prepare and extract the tire particles. For example, Wik and Dave (2006) prepared leachates of fresh tire treads extracted in hard water (hardness = 250 mg/l as CaCO_3_; pH 8.0) at 44°C for 72 h. Acute toxicity to *Daphnia magna* was reported, with 48-h EC_50_s ranging from 500 to >10,000 mg/l among 25 brands of tires tested. In these studies, the researchers established the water hardness and temperatures to specifically inhibit the leaching of some chemicals (i.e., zinc) and encourage the leaching of others (organics). Overall, earlier studies have demonstrated that chemicals in laboratory generated TP may exert toxicity to aquatic biota following extraction or under treatment conditions designed to promote release of those constituents to the aqueous phase. However, the toxicity of TRWP or TP has not been determined under exposure conditions more representative of those expected in the environment; e.g., in the presence of sediment, and ambient temperatures which may affect the availability of chemical constituents in the particles.

The present study was undertaken to address three objectives: (1) to evaluate the acute toxicity of TRWP to several aquatic test species in standardized laboratory tests; (2) to determine whether aqueous extraction of TRWP under high-temperature conditions produces acutely toxic leachate; and (3) to identify the chemical component(s) responsible for acute toxicity in aqueous leachates of TRWP. In initial experiments, acute toxicity to *Pseudokirchneriella subcapitata*, *Daphnia magna*, and *Pimephales promelas* was evaluated using a method in which TRWP was mixed with sediment and water, and the resulting elutriate was tested. Our approach was similar in concept to that described for partially soluble, multi-component substances (i.e., oils, creosote, etc.) where the water-accommodated fractions (WAF) of the substances containing the fraction that is dissolved or present as a stable dispersion/emulsion are assessed (OECD 2000). Such an approach was considered to be a more realistic assessment of potential ecotoxicity than alternative methods such as preparing organic solvent extracts of tire tread. All TRWP constituents that are extractable with water in an environmentally relevant simulated scenario were targeted for this analysis. The inclusion of sediment in the test protocol allowed the bioavailability of the water-extractable constituents to be assessed as well. A second set of toxicity studies were initiated to determine whether aqueous extracts of TRWP (leachates) exhibited toxicity to *D. magna* when prepared using a high-temperature extraction protocol previously shown to result in acute toxicity of tire particle leachates at concentrations less than 5,000 mg/l (Wik and Dave 2006). In these tests, we examined the influence of the temperature at which leachates were prepared, filtering of leachates prior to testing, and the presence of sediment in the test system on the resulting toxicity of the leachate. The results of these experiments were supplemented with a toxicity identification evaluation (TIE) exercise to identify the candidate chemical(s) responsible for acute toxicity in *D. magna* exposed to leachates of TRWP prepared at elevated temperatures.

## Materials and methods

### Tire and road wear particle collection

TRWP were collected at a road simulator laboratory located within the Bundesanstalt für Straßenwesen (BASt), the German Federal Highway Research Institute, as previously described by Kreider et al. (2010). Briefly, the laboratory used an interior drum testing system containing actual asphalt pavement in cassettes. This system was electronically programmable to mimic a variety of driving conditions by varying speed, temperature, acceleration, braking, and steering. For the TRWP collection, the pavement consisted of a standardized asphalt concrete with 6.1% proportion of bitumen (B50/70) according to ISO 10844 (1944). Due to the enclosure around the drum the road surface temperature was maintained at approximately 20°C during the tests. The TRWP was collected using a vacuum system mounted behind one of the simulator wheels. Both summer and winter silica based tires (Michelin Pilot Primacy 225/55 R16 95W and Pirelli Sottozero 225/55 R16 95W M+S) and a carbon-black based summer tire (Bridgestone Potenza RE 88 205/65 R15 94W) were used to generate the TRWP. Particles from each tire were combined to form a single composite (2:1:1 Bridgestone:Michelin:Pirelli) that was sieved at 150 μm to remove any large pavement pieces. The TRWP was shipped in amber glass jars to the toxicity testing laboratory under full chain of custody and were thereafter stored in the dark at 4°C.

### Sediment elutriate toxicity tests

The initial study employed an elutriate approach in preparing the test solutions and an acute water exposure assessment for the toxicity testing. Elutriate designs for preparing test solutions that replicate sediment mobilization and storm water phenomena are recommended and have been successfully employed by other investigators for exposing water column species such as algae and daphnids (ASTM 1990; Baun et al. 2002; Bosch et al. 2009; Novelli et al. 2006).

Freshwater control sediment was collected in November 2007 near the Chualar Bridge on the Salinas River in CA, USA. This location has been used as a reference sediment collection site by the State of California’s Surface Water Ambient Monitoring Program (SWAMP). The sediment was collected using clean stainless steel sampling gear and pre-cleaned HDPE containers. Reference sediment was maintained in a 60-l polyethylene tank in the dark at 4°C under aerated U.S. EPA synthetic moderately hard water. Synthetic water (pH 7.4–7.8; hardness 80–100 mg/l as CaCO_3_; alkalinity 60–70 mg/l as CaCO_3_) was prepared by addition of ACS reagent-grade salts (96 mg/l NaHCO_3_; 60 mg/l CaSO_4_·2H_2_O; 60 mg/l MgSO_4_; 4 mg/l KCl) to Type 1 de-ionized water (U.S. EPA 2002). The control sediment was analyzed for metals (U.S. EPA Methods 6020 m, 245.7 m, 7010), organics (U.S. EPA Method 8270 cm), organotins (Krone et al. 1989), total organic carbon (U.S. EPA Method 9060A), percent solids (U.S. EPA Method 160.3), and grain size. Concentrations of two chlorinated pesticides (4,4-DDE and 4,4-DDT), several metals (arsenic, chromium, copper, lead, mercury, nickel, zinc), and PAHs were greater than detection limits but below ecological health screening limits. Concentrations of all other parameters, including organotins and all PCB congeners, were below detection limits. Total organic carbon was 1.07%, solids were 71.3%, and the grain size measurements indicated sand—30.2–34.5%, silt—53.2–56.3% and clay 12.3–13.5%.

The TRWP elutriate was prepared following standard operating procedures developed prior to test initiation. One-liter aliquots of sediment were transferred into clean 8-l HDPE containers, and 4 l of synthetic moderately hard water were added to each container (sediment:water = 1:4). Aliquots of TRWP were weighed to the nearest milligram and were transferred into the sediment–water mixture to achieve nominal concentrations of 100, 500, 1000, and 10000 mg/l. The contents of the containers were then mixed using stainless steel rotors for 24 h at 22 ± 2°C. After mixing, the contents of each container were allowed to settle for approximately 1 h. Visual examination of the overlying waters after this 1-h settling period indicated that the residual turbidity was still too high for successful testing, so the overlying water was siphoned off and centrifuged in HDPE bottles at 2,500*g* for 15 min. The resulting supernatant comprised the elutriates for these tests. The water quality characteristics of the elutriates (pH, dissolved oxygen, alkalinity, hardness, conductivity, and total ammonia) were within acceptable levels (Table 1).

**Table 1:** Initial water quality characteristics of TRWP-spiked sediment elutriates

Nominal TRWP concentration (mg/l)	pH	Dissolved oxygen (mg/l)	Alkalinity (mg/l)	Hardness (mg/l)	Conductivity (μS/cm)	Total ammonia (mg/l as N)
0	8.16	7.0	110	184	395	3.12
100	8.17	8.8	112	168	519	4.25
500	8.14	7.2	150	208	497	3.53
1,000	8.05	7.1	119	196	463	3.88
10,000	8.03	6.5	110	172	482	2.63

Measured after the centrifugation step, but remained virtually unchanged when the elutriate tests were set up. The elutriate was amended with nutrients prior to algal testing, so the conductivities were ~20% higher in the Selenastrum tests than in the un-amended elutriates; however, the pH levels and DO levels were still very similar

Three control treatments were used for the TRWP testing. Synthetic moderately hard water served as a negative control. Un-spiked sediment elutriate prepared in the same way as spiked elutriate served as a treatment control. Positive controls (reference toxicant tests) consisted of moderately hard water spiked with known concentrations of sodium chloride (U.S. EPA 2002).

Toxicity of elutriates to the alga *Pseudokirchneriella subcapitata* (also commonly referred to as *Selenastrum capricornutum*), the crustacean *Daphnia magna*, and the fathead minnow *Pimephales promelas* was assessed at the Pacific EcoRisk laboratory in Fairfield, CA, USA. Fathead minnows and daphnids were obtained from a commercial supplier (Aquatic Biosystems, Ft. Collins, CO, USA) and maintained in the testing laboratory for an acceptable acclimation period before exposure to the various test conditions. Algal stock cultures on agar were obtained from a commercial collection (University of Texas Culture Collection) and were used to prepare a laboratory culture maintained in log growth phase in algal culture medium. Reference toxicant (NaCl) tests for all three species were performed concurrently with the TRWP test; the resulting test response data were statistically analyzed and compared to the typical response ranges established by previous reference toxicant tests performed in the laboratory. Routine water quality characteristics (temperature, pH, DO, conductivity) were measured on test solutions prior to their use in the tests.

The toxicities of the TRWP elutriates to *P. subcapitata* were determined in 72-h static toxicity tests performed according to *OECD Test Method 201: Alga, Growth Inhibition Test* (OECD 2006). Algae were exposed to TRWP elutriates at nominal test treatment concentrations of 0, 100, 500, 1000, and 10000 mg/l. Twelve replicates of 100 ml test solution were initiated for each test treatment in 250-ml glass flasks; 24 replicates were established for the negative controls. Each flask was inoculated to an initial cell density of 10,000 cells/ml of *P. subcapitata*. Tests were conducted at 24 ± 2°C in continuous cool white fluorescent lighting. Three randomly selected replicates from each treatment were removed daily and algal cell density in each was determined using a hemocytometer; six replicates were analyzed for the laboratory water control treatment. Using regression relationships developed by the testing laboratory immediately prior to the TRWP elutriate testing, the cell density data were transformed into specific growth rates and yield (biomass) data. All test conditions were within acceptable limits during the exposure.

The acute toxicities of TRWP elutriates to *D. magna* were determined in 48-h static testing according to *OECD Test No. 202: Daphnia sp., Acute Immobilisation Test* (OECD 2004). Immobility is used as a surrogate for survivorship and was defined as failure to move within 15 s of gentle agitation of the test container (OECD 2004). Five neonates (<24 h old) per replicate vial (*n* = 4) were exposed to 20 ml of TRWP elutriates at concentrations of 100, 500, 1000, and 10000 mg/l (in addition to the lab water control and sediment elutriate control) for 48 h, after which effects on mobility were evaluated. The *D. magna* were maintained at 21 ± 2°C under a 16 h light:8 h dark photoperiod.

Acute 96-h toxicity tests with fathead minnows adhered to OECD Test No. 203: Fish, Acute Toxicity Test (OECD 1992). Seven larval (9 days old) fish per replicate (*n* = 4) were exposed to TRWP elutriates at 100, 500, 1000, and 10000 mg/l (in addition to the lab water control and sediment elutriate control) for 96 h at 21 ± 2°C in a 16 h:8 h light/dark photoperiod. Organisms were examined daily and mortalities in each replicate were determined.

### Toxicity of TRWP leachate

A second round of testing was conducted to investigate the chemical constituents of TRWP in an aqueous phase (“leachate”) prepared under an elevated temperature regimen. Four treatment regimens were tested.

Treatments 1 and 2 consisted of aqueous leachates prepared according to a previously published study (Wik and Dave 2006). An aliquot of TRWP was weighed out to the nearest milligram and added to a 600-ml glass beaker containing 300 ml of synthetic moderately hard water, to achieve a nominal concentration of 10,000 mg/l TRWP. A beaker of water without TRWP served as the leachate blank. The contents of the beakers were mixed using glass pasteur pipets and covered with aluminum foil. In treatment 1, the beakers and contents were incubated for 72 h in an oven at 44°C then allowed to cool to room temperature, while in treatment 2, the beakers and contents were incubated in darkness at room temperature (21°C) for 72 h. Following incubation, the leachates were filtered through sterile pre-rinsed Nalgene 0.45-µm filters under vacuum. The resulting filtrates comprised the 100% leachates for Treatments 1 and 2. Nominal TRWP concentrations of 625, 1250, 2500, and 5000 mg/l were prepared from these leachates by serial dilution using moderately hard water.

Treatments 3 and 4 consisted of leachates mixed with sediment. Leachates were prepared as described above. Following the 72-h incubation at 44°C (Treatment 3) or 21°C (Treatment 4), leachates were added to reference (unspiked) sediment (1:4 sediment:leachate) and mixed using stainless steel rotors for 24 h then allowed to settle for 1 h. The elutriates were decanted and filtered through sterile pre-rinsed Nalgene^®^ 0.45-μm filters. The resulting filtrates comprised the 100% leachates for Treatments 3 and 4. Test solutions at nominal TRWP concentrations of 625, 1250, 2500, and 5000 mg/l were prepared by serial dilution of these leachate elutriates using moderately hard water.

Acute toxicity of leachates was tested using the OECD 48-h *D. magna* survival test (OECD 2004) as described previously. Three replicate vials were prepared for each treatment and control. The test was terminated after 48 h.

### Toxicity identification evaluation

Phase 1 TIE manipulations were performed according to U.S. EPA guidelines (U.S. EPA 1991). Two rounds of TIE were completed. In the initial round of TIE, a 100% leachate and a 50% leachate (in addition to control water) were subjected to a series of manipulations and tested for acute toxicity using the 48-h *D. magna* test, as described previously (three replicates were prepared for each treatment and control). A second round of testing was conducted to confirm initial results and further resolve constituents responsible for observed toxicity. Owing to a limited amount of TRWP remaining after the initial TIE, the follow-up TIE was limited to testing 100% leachate only.

TRWP leachate was prepared by incubating 10,000 mg/l TRWP in moderately hard water at 44°C for 72 h as previously described for leachate toxicity tests. The TRWP leachate and the blank were vacuum-filtered through pre-rinsed Nalgene^®^ sterile 0.45 μm filters. The resulting filtrate comprised the 100% leachate that was used in the acute *D. magna* toxicity test. The 50% leachate was prepared by dilution using moderately hard water.

Subsamples of the 100% leachate collected following the 44°C treatment but prior to TIE manipulations or toxicity testing were analyzed for metals, PAHs, and select organic compounds present in tires as antioxidants, accelerators, vulcanizing agents, or contaminants. Metal concentrations were quantified by Enviromatrix, San Diego, CA using ICP-MS (EPA 200 series methods), and PAHs by GC–MS (EPA Method 8270C). Organic chemicals were analyzed by Akron Rubber Development Laboratory, Akron, OH using identification and quantification with LC/MS/MS.

Phase I TIE manipulations were designed to identify major classes of compounds (organics, metals, ammonia, etc.) responsible for leachate toxicity. Because the quantity of TRWP was limited, a targeted Phase 1 TIE consisting of selected TIE treatments was performed rather than the full complement of TIE treatments. A treatment blank consisting of synthetic moderately hard water was subjected to each manipulation concurrent with TRWP extracts. A baseline toxicity test of untreated leachate was also conducted concurrent with each manipulation to serve as a reference benchmark against which toxicity removal by the other TIE treatments was assessed. Ethylenediaminetetraacetic acid (EDTA) at 40 μM was used to characterize toxicity due to metals that are amenable to chelation by EDTA. Sodium thiosulfate (STS) at 60 μM was used to characterize toxicity due to residual chlorine; however, STS is also effective at removing the toxicity of some metals. In conjunction with patterns of toxicity removal by EDTA, STS can facilitate a better resolution of specific metals causing toxicity. A cation exchange solid phase extraction (SPE) treatment (Chelex^®^ 100, Bio-Rad, Hercules, CA, USA) was used to identify extract toxicity due to cationic compounds, including many metals. An appropriate volume of leachate was passed over Chelex SPE columns and the treated extract was collected. Because the Chelex treatment also effectively removes calcium and magnesium (the primary elements of “hardness”), the Chelex-treated leachate was the amended with calcium chloride and magnesium chloride to bring the calcium and magnesium back up to the concentrations measured in the untreated TRWP leachate prior to testing. An anion exchange treatment was used to identify leachate toxicity due to anionic compounds. An appropriate volume of leachate was passed over an AG 1-8X SPE column (Bio-Rad, Hercules, CA, USA) and the treated leachate was collected and tested to assess any effects on the toxicity. Leachates were passed over a C_18_ SPE column (J.T. Baker, Phillipsburg, NJ, USA) to remove non-polar organic compounds and some relatively non-polar metal chelates. The treated leachate was then tested to assess any effects on the toxicity. The C_18_ SPE column was subsequently eluted with methanol, the eluate was diluted to the initial leachate concentration (1×) with control water (the concentration of methanol in the final reconstituted sample was ~0.3%), and the 1× eluate (and accompanying method blank) was then tested to assess recovery of toxicity. In the second round of TIE, the leachate sample was also passed over a hydrophilic DVB SPE column (J.T. Baker, Phillipsburg, NJ, USA) to remove polar organic compounds. The DVP-treated leachate (and accompanying method blank) was then tested to assess any effects on the toxicity.

### Data analysis

All statistical analyses were performed using CETIS^®^ (Comprehensive Environmental Toxicity Information System) statistical software (TidePool Scientific, McKinleyville, CA, USA). Data were tested for normality and homogeneity using the Modified Levene, Shapiro–Wilk, and Bartlett tests as appropriate. All tests were performed at least to the *p* = 0.05 level of significance. For the algal growth toxicity tests, data analysis involved using regression relationships developed at the Pacific EcoRisk lab (with *r* ^2^ > 0.98 for each regression). Algal cell density data were transformed into specific growth rates (logarithmic increase of biomass per day) and yield (biomass at the end of the test minus the starting biomass), and data were analyzed to determine key concentration–response point estimates (IC_50_) using linear interpolation methods. No observed adverse effect levels (NOAEL) and lowest observed adverse effect levels (LOAEL) were calculated using Bonferroni, Steel’s Many-One Rank, and Dunnett’s Multiple Comparison tests. Statistical methods employed for the acute daphnid survival test included Trimmed Spearman–Karber, Steel’s Many-One Rank, Bartlett, and Shapiro–Wilk tests. Point estimates for the 96 h survival of fathead minnows were calculated using linear regression, and Steel’s Many-One Rank was employed for the NOAEL and LOAEL estimates.

## Results

### Acute toxicity of TRWP sediment elutriate

Growth of *P. subcapitata* and survival of *D. magna* and fathead minnows in control water were within acceptable ranges. Healthy cell counts in algal controls increased exponentially at least by a factor of 16 (equivalent to at least 0.92 growth rate per day) within the 72-h test period, thereby satisfying the OECD test acceptance criteria. Results of the reference toxicity tests with NaCl (algae IC_50_ = 1.29 g/l; daphnid EC_50_ = 4 g/l; fathead LC_50_ = 4.8 g/l) were consistent with previous reference toxicity tests performed at Pacific EcoRisk, indicating that these organisms were responding in a typical fashion.

Spiked sediment elutriates of TRWP exhibited no acute toxicity in alga, *D. magna*, or fathead minnows (Table 2). No concentration-dependent responses were observed in any of the organisms. As a result, the highest concentration tested at 10,000 mg/l was established as the NOAEC for all endpoints in all three organisms (Table 3). Neither effect concentration (EC_50_) for daphnids and algae, nor lethal concentration (LC_50_) for fish, could be estimated, but are greater than 10,000 mg/l.

**Table 2:** Acute toxicity of elutriates of sediment spiked with TRWP to mean (*n* = 3) specific growth rate of the green alga *P. subcapitata*, mean (*n* = 4) 48-h survival of *D. magna*, and mean (*n* = 4) 96-h survival of *P. promelas*

Nominal TRWP concentration (mg/l)	*P. subcapitata* specific growth rate, 0–72 h (day^−1^)	*D. magna* survival, 48 h (%)	*P. promelas* survival, 96 h (%)
0	1.44 (0.071)	100	100
100	1.54 (0.0139)	100	100
500	1.50 (0.0156)	100	100
1,000	1.52 (0.0292)	100	100
10,000	1.47 (0.0439)	100	100

Values in parentheses represent standard deviation of the mean

**Table 3:** No-effect concentrations and median effect concentrations for aquatic species exposed to sediment elutriates of TRWP or aqueous leachates of TRWP

Organism/endpoint	Treatment	NOAEC (mg/l)	LC/EC_50_^a^ (mg/l)
*P. subcapitata*, 0–72 h growth rate	Sediment elutriate	>10,000	>10,000
*P. promelas*, 96-h survival	Sediment elutriate	>10,000	>10,000
*D. magna*, 48-h survival	Sediment elutriate	>10,000	>10,000
*D. magna*, 48-h survival	Leachate, 44°C	1,250	4,360 (3,660–5,250)
*D. magna*, 48-h survival	Leachate, 21°C	>10,000	>10,000
*D. magna*, 48-h survival	Leachate + sediment, 44°C	2,500	5,080 (4,250–6,070)
*D. magna*, 48-h survival	Leachate + sediment, 21°C	>10,000	>10,000

All treatments were exposed to nominal concentrations of TRWP up to 10,000 mg/l

^a^LC_50_ is used for *P. promelas*, while EC_50_ refers to *P. subcapitata* and *D. magna*. The 95th LCL-UCL is shown in parenthesis

### Acute toxicity of TRWP leachate

**Fig. 1:**
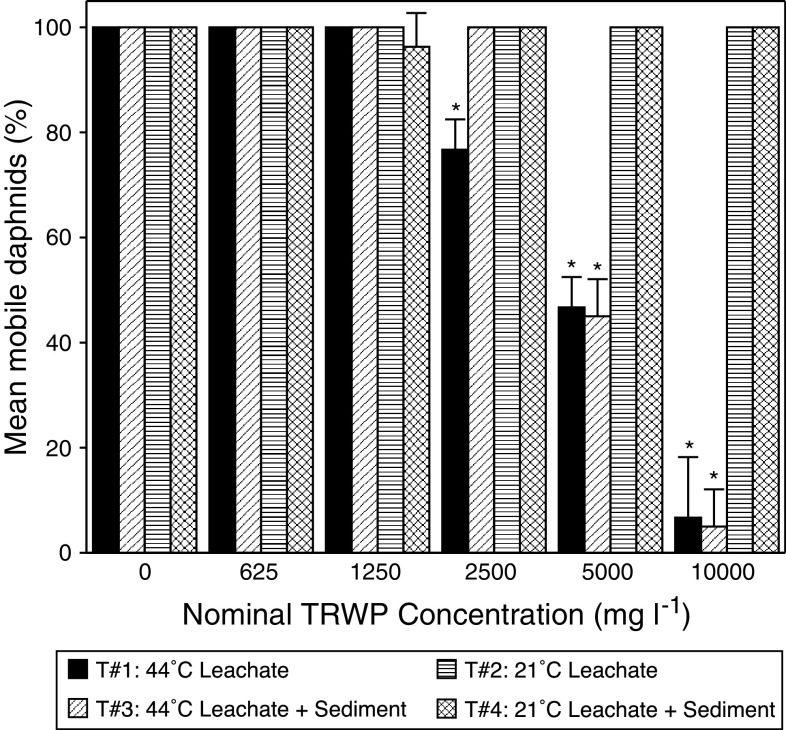
Mean (*n* = 4) survival of *D. magna* after 48 h exposure to aqueous leachates of TRWP extracted at 44°C (Treatment 1) or 21°C (Treatment 2), or sediment elutriates of TRWP incubated at 44°C (Treatment 3) or 21°C (Treatment 4). *Error bars* represent standard deviation of the mean. *Asterisks* indicate significantly different from 0 mg/l control treatment (*p* < 0.05)

Aqueous leachate prepared by incubating TRWP in water at 44°C for 72 h was acutely toxic to *D. magna*, but leachates prepared at room temperature were not. Leachate incubated at 44°C (Treatment 1) resulted in a concentration-dependent decrease in survival of *D. magna*; whereas leachate incubated at room temperature (Treatment 2) did not (Fig. 1). An EC_50_ of 4,360 mg/l and a NOAEC of 1,250 mg/l were calculated for TRWP extracted at 44°C (Table 3); due to the absence of impairment, equivalent values could not be calculated for aqueous extracts incubated at ambient temperatures.

The presence of sediment appeared to have only a mild effect on toxicity to *D. magna* of TRWP leachate. Leachate incubated at 44°C for 72 h and subsequently mixed with sediment (Treatment 3) resulted in a concentration-dependent decrease in *D. magna* survival similar to that observed for Treatment 1, the 44°C aqueous extract without sediment (Fig. 1). An EC_50_ of 5,080 mg/l and a NOAEC of 2,500 mg/l were estimated for the leachate incubated at 44°C and mixed with sediment (Table 3). TRWP leachates incubated at room temperature, with or without sediment, were not toxic to *D. magna* (Fig. 1).

### Toxicity identification evaluation

Baseline toxicity testing in the initial phase of TIE testing confirmed leachate prepared at 44°C was acutely toxic to *D. magna*: 100% leachate reduced survival from 93 to 53% (Table 2). Addition of 40 μM EDTA had only a minor effect and did not remove toxicity (40% survival in 100% leachate). The addition of 60 μM STS resulted in a slight increase of toxicity, with only 27% survival in *D. magna* exposed to 100% leachate. Passing the leachate through the cation exchange column (Chelex) substantially increased toxicity, with no *Daphnia* surviving at 100% leachate and modestly lower survival at 50% leachate (Table 4). Passing leachate samples through an anion exchange column and the C18 SPE resin both resulted in a slight increase in toxicity. Toxicity was recovered in the eluate from the C18 resin.

**Table 4:** Survival of *D. magna* (%) in TRWP leachates subjected to TIE

Treatment	Blank	50% Leachate	100% Leachate	Effect on toxicity	Chemical removed by treatment
Baseline	93.3	93.3	53.3	–	–
40 μM EDTA	93.3	100	40	No removal of toxicity	Metals
60 μM STS	93.3	53.3	26.7	Slight increase in toxicity	Chlorine, metals
Cation exchange	100	86.7	0	Increased toxicity	Cationic compounds
Anion exchange	100	40	26.7	Slight increase in toxicity	Anionic compounds
C18 SPE	100	53.3	33.3	Slight increase in toxicity	Non polar organics
C18 eluate	93.3	86.7	33.3	Toxicity recovery	–

Data from the initial round of TIE manipulations are shown. Treatments were addition of 40 μM EDTA, addition of 60 μM STS, cation resin exchange, anion resin exchange, C18 SPE, and elution of the C18 SPE column

In the second round of TIE testing, different manipulations were applied to 100% leachate to resolve the nature of the agent(s) responsible for acute toxicity in *D. magna*. As with the previous test, passing the leachate through a cation exchange column resulted in increased toxicity, with no survival (Table 5). Application of the DVB SPE column resulted in complete removal of toxicity. Application of both the cation exchange and DVB SPE manipulations also resulted in complete removal of toxicity. In contrast with the initial round of TIE, the C18 SPE manipulation also removed toxicity, with 93% *Daphnia* survival. The combination of cation exchange and the C18 SPE treatment caused a slight increase in toxicity, with 17% survival of *Daphnia*.

**Table 5:** Survival of *D. magna* (%) in TRWP leachates subjected to TIE

Treatment	Blank	100% Leachate	Effect on toxicity
Baseline	100	26.7	–
Cation exchange	100	0	Increased toxicity
DVB SPE	100	100	Complete removal
Cation exchange + DVB SPE	100	100	Complete removal
C18 SPE	100	93.3	Complete removal
Cation exchange + C18 SPE	100	16.7	Slight increase

Data from the second round of TIE manipulations are shown. Treatments were addition of 40 μM EDTA, addition of 60 μM STS, cation resin exchange, anion resin exchange, C18 SPE, and elution of the C18 SPE column

## Discussion

Elutriates of TRWP-spiked sediment exhibited no toxicity to three common aquatic test species at nominal concentrations of 10,000 mg/l or less. It may be inferred from these results that TRWP, even at high concentrations in the aquatic environment, are unlikely to pose an acute toxicity hazard. The results of the alga test, which is a multigenerational evaluation, also suggest that there is a decreased likelihood of chronic toxicity, and testing is currently underway to specifically evaluate the chronic toxicity potential of TRWP. In natural aquatic systems containing sediment and suspended particulates, sediment acts as a sink for many chemicals with poor aqueous solubility and, as a result, the bioavailability of contaminants is greatly diminished. The rationale for the elutriate testing was that it more accurately represented environmental conditions where TRWP (and potential leachate) are: (1) washed via advective transport off roadway surfaces into storm water flow that picks up solids as it is conveyed into receiving streams to be eventually stored in sediment; and (2) resuspended, released, and remobilized from sediment into the water column by increases of water flow. Based on exposure concentrations of TRWP employed in the present study, it appears that when TRWP are exposed to aquatic sediments, subsequent mixing of the sediments with water (as in a resuspension event) is unlikely to cause significant short-term effects on growth, mobility, and survival in aquatic test species even at concentrations as high as 10,000 mg/l.

The lack of toxicity of TRWP sediment elutriates and leachates prepared at ambient temperatures contrasts with the findings of Wik et al. (2009), who reported lower EC_50_ values in *P. subcapitata*, *D. magna*, and *Ceriodaphnia dubia* exposed to a series of sequential aqueous extracts of tire powder abraded from tire treads using a rasp. Both chronic and acute endpoints were assessed; the lowest effect concentrations were found for chronic endpoints. Under the conditions used in their tests, nine-day reproduction in *C. dubia* was the most sensitive endpoint, with an EC_50_ of 10 mg/l from the most toxic tire. Tire leachates exhibited acute toxicity in *D. magna* at somewhat higher concentrations, with EC_50_s for 48-h immobility ranging from 370 to 7,450 mg/l depending on tire composition and number of extractions. In *P. subcapitata*, EC_50_s for 72-h growth ranged from 50 to 2,840 mg/l. *Danio rerio* (Zebra fish) eggs were also exposed to tire particles, but no consistent toxicity was found at leachate concentrations up to 10,000 mg/l. Thus, for some of the tires tested, the lower acute toxicity to these organisms was similar to results found in our tests with TRWP. However, in general, lower EC_50_s were reported for tire particles compared to those found for TRWP. Wik et al. (2009) did not provide complete details of the extraction methods used (e.g., the temperature at which treads were extracted); nevertheless, it is apparent that differences between Wik’s results and our findings can be attributed, in large part, to differences in extraction methods and test conditions. In particular, Wik et al. tested tire particles rather than TRWP, which better represent the particles expected to enter roadside streams. The use of sediment elutriates and ambient temperatures in our tests are considered to be more representative than the aqueous extracts of tire particles tested by Wik et al. for predicting effects in actual stream or pond systems containing a sediment sink for tire wear particles.

Previous investigations of TP toxicity have employed extraction conditions designed to maximize extraction of tire constituents to the aqueous phase. Gualtieri et al. (2005b) extracted TPs in water at pH 3 to simulate a “worst-case” scenario in water bodies affected by acid precipitation; 100% extracts at 50,000 mg/l were lethal to *D*. *magna* in 24- and 48-h exposures, and inhibited growth of the alga *P. subcapitata*. Mortality and malformations in *Xenopus laevis* embryos exposed to tire particles extracted in acidic water or in dichloromethane were also noted (Gualtieri et al. 2005a). Extraction of tire material at higher temperatures has also been shown to promote the release of toxic constituents. Wik and Dave (2006) prepared leachates of fresh tire treads extracted for 72 h at 44°C in hard water (hardness = 250 mg/l as CaCO_3_; pH 8.0). Acute toxicity to *D. magna* was reported, with 48-h EC_50_s ranging from 500 to 5,000 mg/l among 25 brands of tires tested.

The results of the current leachate toxicity test confirm that strong extraction conditions such as high-temperature incubation also can leach constituents from TRWP that are acutely toxic to *D. magna*. TRWP appears to be somewhat less toxic to *D. magna* than TPs from most tires tested by Wik and Dave (2006) extracted under the same high-temperature conditions; the EC_50_ value of 4,360 mg/l for TRWP was similar to that for TPs from the most toxic tire, but greater than most of those reported by Wik and Dave (mean 48-h EC_50_ = 2 g/l). Importantly, toxicity to *D. magna* was ameliorated somewhat by the introduction of sediment to the system after the high-temperature extraction. The observation of lower toxicity suggests that some fraction of the toxicants released by the high-temperature extraction were bound to sediment and unavailable to *D. magna*.

Previous investigations have implicated metals and organic compounds in tire particles as potential toxicants (Gualtieri et al. 2005b; Nelson et al. 1994; Wik and Dave 2006; Wik et al. 2009). The results of the two TIEs conducted on high-temperature leachates of TRWP suggest the presence of two co-occurring contaminants, one a cation (e.g., metal) and the other an anionic organic compound, and that there is an antagonistic interaction between these contaminants. In the first round of TIE, the increase in toxicity resulting from the STS and Chelex treatments indicates that metals are playing a role in the toxicity, but these treatments increased the toxicity instead of the more typical reduction in toxicity. Toxicity was recovered in the C18 eluate, suggesting that an organic contaminant that had sorbed to the C18 resin was contributing to toxicity. However, C18 SPE increased the toxicity instead of the expected accompanying reduction in toxicity (as was suggested by toxicity recovery in the C18 eluate). The results together suggested the presence of co-occurring contaminants, one a metal or other cationic contaminant, and the other an organic compound, acting antagonistically to each other. The increase in toxicity observed for the anion exchange treatment was consistent with the hypothesis of co-occurring contaminants with antagonistic toxicity, and suggested the presence of a separate anionic contaminant, or that the organic contaminant was anionic in nature. The results of the second round of TIE testing were consistent with initial results, in that the cation exchange treatment removed the cationic contaminant, and the toxicity of the remaining organic contaminant increased. Moreover, the DVB treatment (intended to remove polar organic chemicals) removed the toxicity, suggesting that the antagonistic interaction effect is one-way: from the cation to the organic chemical. Assuming that the DVB treatment effectively removed the organic component, then additional cation exchange treatment (Cation + DVB) could no longer increase the toxicity via antagonistic interaction, as the organic contaminant had been completely removed. On the other hand, partial toxicity in the cation exchange plus C18 SPE treatment suggests incomplete removal of the organic component, allowing the remaining organic component to then exhibit its remaining partial toxicity.

The TIE results are consistent with previous investigations suggesting the presence of multiple toxicants in tire particle leachates (Abernethy 1994; Gualtieri et al. 2005b; Wik and Dave 2009). Chemical analysis of the heated leachate revealed some candidate chemicals (Table 6). Among cations analyzed, zinc is the most likely to be exhibiting toxicity. Zinc is the most abundant heavy metal in tires, and was present in the heated leachate at a concentration (56 μg/l) that may contribute to acute toxic effects in *D. magna* but is unlikely to be the dominant toxicant. Acute lethal concentrations of soluble zinc in *D. magna* 48-h exposures range from approximately 50 μg/l to >3 mg/l depending on test conditions (Mount and Norberg 1984; Muyssen and Janssen 2007; Oda et al. 2006) and the Species Mean Acute Value for *D. magna* reported by the U.S. EPA is 355.5 μg/l (U.S. EPA 1996). Several of the organic chemicals used in tires as antioxidants and vulcanizing agents were detected in the heated leachate sample; however, only two were present at quantifiable levels: aniline (16 mg/l), and *N*,*N*′-bis(1,4-dimethylpentyl)-*p*-phenylenediamine (77PD; 26 mg/l). Aniline has been shown to be acutely toxic to *D. magna* at concentrations much lower than those found in the leachate. Median lethal values (LC_50_) at 48 h exposure range from 40 to 680 μg/l (Canton and Adema 1978; Maas-Diepeveen and Van Leeuwen 1986). Thus, aniline is a strong candidate for the organic component of toxicity in heated TRWP leachate. Aniline as the organic toxicant is also consistent with the TIE results; as a polar organic chemical, aniline would be effectively removed by the DVB SPE treatment to a greater degree than C18 SPE.

**Table 6:** Chemical analysis of heated TRWP leachate

Parameter	Leachate blank (mg/l)	TRWP Leachate (mg/l)
Arsenic	<0.0001	0.016
Calcium	16.6	27.1
Cadmium	<0.0002	<0.0002
Chromium	<0.0002	0.0004
Copper	<0.0002	<0.0002
Lead	<0.0001	<0.0001
Magnesium	13.1	14.8
Mercury	<0.00008	<0.00008
Nickel	<0.0002	0.005
Potassium	2.51	8.42
Silver	<0.0002	<0.0002
Sodium	31.1	36.6
Zinc	0.0007	0.056
Acenaphthene	<0.00077	<0.00077
Acenaphthylene	<0.00087	<0.00087
Anthracene	<0.00063	<0.00063
Benzo(a)anthracene	<0.000550	<0.000550
Benzo(b)fluoranthene	<0.00180	<0.00180
Benzo(k)fluoranthene	<0.00139	<0.00139
Benzo(g,h,i)perylene	<0.00109	<0.00109
Benzo(a)pyrene	<0.000650	<0.000650
Chrysene	<0.000500	<0.000500
Dibenz(a,h)anthracene	<0.000950	<0.000950
Fluoranthene	<0.000600	<0.000600
Fluorene	<0.000550	<0.000550
Indeno(1,2,3-cd)pyrene	<0.000990	<0.000990
Naphthalene	<0.00161	<0.00161
Phenanthrene	<0.000460	<0.000460
Pyrene	<0.00115	<0.00115
2-Mercaptobenzothiazole	<1.7	<1.7
Dicyclohexylamine	<0.3	<0.3
2,2,4-Trimethyl-1,2-dihydroquinoline	<1.7	<1.7
Resorcinol	<5	<5
Aniline	<1.7	16
Butyl hydroxytoluene (BHT)	<1.7	<1.7
2,2-Methylene-bis-(4-methyl-6-*tert*-butylphenol) (BPH)	<0.02	0.007–0.02^a^
*N*,*N*′-Diphenyl-*p*-phenylenediamine (DPPD)	<5	<5
*N*-1,3-Dimethyl-butyl-*N*′-phenyl-*p*-phenylenediamine (6PPD)	<1	<0.3
*N*-Isopropyl-*N*′-phenyl-*p*-phenylenediamine (IPPD)	<1	<0.3
*N*,*N*′-Bis(1,4-dimethylpentyl)-*p*-phenylenediamine (77PD)	1.7–5^a^	>38
Diphenylamine	<1.7	<1.7

TRWP were incubated with moderately hard water at 44°C for 72 h, and the resulting leachate was analyzed for dissolved metals, PAHs, and select organic constituents

^a^Detected but not quantifiable; range represents range of limit of detection and limit of quantification

Although uncommon, antagonism between chemicals such as metals, fungicides, surfactants and pesticides has been reported in a number of aquatic toxicity studies (Weis and Weis 1987; Ozoh 1980; Voyer et al. 1982; Bowers et al. 1980; Hernando et al. 2005; Norgaard and Cedergreen 2010; Rosal et al. 2010). Even in chemicals with known, similar mechanisms of action, antagonism has been reported. For example, slight antagonism was found after *Hyalella azteca* was exposed to two pyrethroid pesticides. As the mechanism of toxicity of these two pesticides is similar, the result was unexpected but may be due to differences in metabolism of the pesticides (Brander et al. 2009). While no studies were identified that specifically found antagonism between a metal and organic, based on the published literature, it appears that the antagonistic results observed in this study are plausible.

## Conclusion

The results from the sediment elutriate exposure study indicated low potential for risk from exposure to tire wear particles in aquatic ecosystems. TRWP were not acutely toxic to any species tested in short term exposures, with EC_50_ values all exceeding 10,000 mg/l. These results suggest that under conditions approximating those in which TRWP are likely to enter the environment, TRWP do not release chemicals at concentrations capable of causing acute effects in aquatic receptors, and that TRWP should be considered a low toxicity concern to aquatic ecosystems based on acute exposure scenarios. The conclusions of leachate toxicity tests demonstrated that harsh conditions such as high-temperature can cause the release of toxic constituents from TRWP, but such conditions are not considered to be representative of conditions in aquatic environments receiving TRWP. TIEs of TRWP leachate revealed the presence of a cation and a polar organic compound acting antagonistically. Based on chemical analysis of the leachate, zinc and aniline were identified as the most likely candidate toxicants.
