# Correction to: Acute aquatic toxicity of tire and road wear particles to alga, daphnid, and fish

**DOI:** 10.1007/s10646-020-02228-x

**Published:** 2020-05-19

**Authors:** Christopher Marwood, Britt McAtee, Marisa Kreider, R. Scott Ogle, Brent Finley, Len Sweet, Julie Panko

**Affiliations:** 1 ChemRisk Canada, 291 Woodlawn Road West, Guelph, ON N1H 7L6 Canada; 2 ChemRisk, 20 Stanwix Street, Suite 505, Pittsburgh, PA 15222 USA; 3 Pacific EcoRisk, Inc., 2250 Cordelia Road, Fairfield, CA 94534 USA; 4 ChemRisk, 25 Jessie Street, Suite 1800, San Francisco, CA 94105 USA; 5 Present Address: NovaTox Inc., 10 Crane Avenue, Guelph, ON N1G 2R2 Canada; 6 Present Address: School of Public Health, University of Michigan, 1415 Washington Heights, Ann Arbor, MI 48109 USA

Correction to: Ecotoxicology (2011) 20:2079–2089

10.1007/s10646-011-0750-x

The article Acute aquatic toxicity of tire and road wear particles to alga, daphnid, and fish, written by Christopher Marwood, Britt McAtee, Marisa Kreider, R. Scott Ogle, Brent Finley, Len Sweet, and Julie Panko, was originally published electronically on the publisher’s internet portal on 26 July 2011 without open access. With the author(s)’ decision to opt for Open Choice the copyright of the article changed on May 2020 to © The Author(s) 2020 and the article is forthwith distributed under a Creative Commons Attribution 4.0 International License (https://creativecommons.org/licenses/by/4.0/), which permits use, sharing, adaptation, distribution and reproduction in any medium or format, as long as you give appropriate credit to the original author(s) and the source, provide a link to the Creative Commons licence, and indicate if changes were made.

**Publisher’s Note** Springer Nature remains neutral with regard to jurisdictional claims in published maps and institutional affiliations.

